# Editorial: Innovative adjuvant strategies: enhancing vaccine efficacy through transdisciplinary approaches

**DOI:** 10.3389/fimmu.2026.1918784

**Published:** 2026-08-07

**Authors:** Carlos Angulo, Alejandro Daniel Parola, Abel A. Ramos Vega

**Affiliations:** 1 Northwestern Center of Biological Research, S.C. (CIBNOR), La Paz, Mexico; 2 Fundación Pablo Cassará, Ciudad de Buenos Aires, Argentina; 3 Centro de Investigación en Ciencia Aplicada y Tecnología Avanzada (CICATA) Unidad Morelos del Instituto Politécnico Nacional (IPN), Xochitepec, Mexico

**Keywords:** adjuvants agents, health, immunogenicity, protective efficacy, vaccines

Adjuvants play a crucial role in amplifying vaccine efficacy to prevent and control diseases. They can also improve patient comfort and well-being, for instance, by reducing the required dosage or number of vaccine doses, making them an affordable strategy for improving accessibility and achieving global health equity. Through historical scientific efforts, several adjuvants have been designed, tested, and approved for animals and humans. However, drawbacks related to the safety, consistency, and scalability of novel adjuvants impede their global implementation for the benefit of humankind. This research topic presents recent experimental findings and a conceptual framework that enhances our understanding of the design and applicability of innovative adjuvants.

For instance, infants are highly susceptible to infectious diseases, especially during the neonatal period. Molina Estupiñan et al. offer a clear example of how novel adjuvants based on enterotoxins (dmLT and mmCT) and cationic liposomes (CAF01 and CAF08b) can reduce the dose needed to achieve protective efficacy against *Streptococcus pneumoniae* and influenza virus infections in a neonatal mouse model compared to the alum (aluminum hydroxide) adjuvant. These findings provide insight into adjuvant research for early-life vaccinations, reducing the amount of vaccine needed while offering the opportunity to increase coverage during outbreaks. Moreover, efficacy can also be improved depending on the immunization strategy, as demonstrated by (Fourutan Pajoohian et al.). Neonatal mice were immunized against pneumonia using a conjugate vaccine with a CAF01 adjuvant via heterologous subcutaneous (s.c.) priming, followed by an intranasal (i.n.) booster with a mmCT adjuvant or homologous immunizations via either route. The results indicate that heterologous s.c./i.n. immunizations also induced high antibody responses, avoiding the need for a second invasive injection in neonates, which could provide a clear clinical benefit.

The development of nanoadjuvants is one of the most dynamic frontiers in vaccine innovation, integrating delivery systems with immune potentiation. In the study by Rizzo et al., a novel polyallylamine-based self-assembled nanoparticle, encapsulating ovalbumin as a model antigen, was administered intranasally and intraperitoneally in mice. *In vitro* and *in vivo* experiments evidenced phagocytic uptake, pro-inflammatory cytokine secretion via the canonical NLRP3-caspase-1 inflammasome, and Th1 immune responses. Overall, this study supported the efficacy and versatility of this nanoadjuvant in inducing mucosal and systemic immune responses in animals immunized parenterally and mucosally. In line with this topic, innovative nanodiamonds loaded with a plant-made recombinant antigen from the Porcine epidemic diarrhea virus (PEDV) were studied as an adjuvanted vaccine (Ho et al.). Pregnant sows immunized intramuscularly produced specific IgG (blood) and IgA (milk) antibodies, and piglets from immunized mothers had virus-neutralizing antibodies. The authors emphasized that plant-made recombinant antigen technology can be combined with nanotechnology to advance PEDV vaccine development.

Food-grade vaccines are an alternative strategy for oral vaccination that minimizes the stress of parenteral immunizations. The Gram-positive enhancer matrix (GEM) is derived from bacteria and is devoid of intracellular contents and native surface proteins while retaining an intact peptidoglycan cell wall envelope. Based on this approach, Zhang et al. displayed the surface-anchoring protein and the M cell-targeting peptide (SAM) with a *Helicobacter pylori* multi-epitope antigen (FVpE) in the GEM of *Lactococcus lactis*. Oral administration to mice induced mucosal and systemic antibody production, along with Th1, Th2, and Th17 responses, conferring protection against a *Helicobacter pylori* challenge. The use of other microbial components as adjuvants has also been investigated. Could β-glucans enhance the immune response to the SARS-CoV-2 vaccine by inducing trained immunity and boosting neutralizing antibody production? This is the question addressed in the study by (Golim et al.). The answer: In a clinical trial, volunteers who received β-1,3-1,6-glucan from *Saccharomyces cerevisiae* and the ChAdOx1 nCoV-19 vaccine (two doses) produced higher neutralizing anti-SARS-CoV-2 antibodies than the placebo group. These results advance the applicability of oral β-glucans as adjuvants for COVID-19 vaccines by augmenting the specific humoral immune response. In another clinical trial, the immunogenicity of HIV vaccines was compared with MF59, Alum, and AS01B adjuvants. Menezes et al. found that the AS01B adjuvant had superior immunogenicity against several antigens, and that this was characterized by elevated IgG-binding antibodies and CD4+ T-cell responses without serious adverse events. These results reinforce the rationale behind the future of preventive HIV vaccines with the appropriate adjuvant and merit further investigation.

The link between innate and adaptive immune responses to vaccine adjuvants is of interest to many researchers. This is the case of Carvalho et al., who produced a recombinant BCG expressing a detoxified *Escherichia coli* heat-labile toxin subunit and tested it as an adjuvanted vaccine *in vitro* and in mice. Primed macrophages showed inflammasome activation mechanisms linked to CD4+ T cell function and Th1/Th17 responses. This type of study is important as it provides clues about other unknown potential adjuvants. One example is the search for immunostimulatory molecules in aquatic organisms. Chiumiento et al. reported that the hemocyanin from the freshwater snail *Pomacea canaliculata* induced proinflammatory cytokine production and morphological, functional, and metabolic effects in monocyte-macrophages. Mice immunized with hemocyanin produced specific antibodies, which led to the hypothesis of induction of a cytotoxic (Th1) immune response. Another approach to searching for novel adjuvants and studying the innate-adaptive response axis involves chemically synthesizing small compounds. Yokoyama et al. demonstrated that a small molecule, identified as 2G272 (ethyl 2-(benzo[c][1,2,5]selenadiazole-4-sulfonamido)-4,5-dimethylthiophene-3-carboxylate), in combination with ovalbumin, induced a Th2 immune response in mice, as evidenced by IgG1-specific antibody production. This response was associated with enhanced dendritic cell function dependent on calcium influx.

Advancements in artificial intelligence have impacted the immunoinformatic approach for vaccine and adjuvant design. Ji et al. developed a machine-learning workflow to predict antibody production upon vaccination using a 9-valent HPV vaccine adjuvant and non-human primate RNA transcriptomic data. Accurate and robust predictions can identify adjuvant types and the magnitude of immune responses, thereby facilitating adjuvant selection. This approach provides a foundation for the design of recombinant adjuvants and will likely be part of any adjuvanted vaccine platform in the near future.

This Research Topic also includes four comprehensive reviews that contextualize current knowledge of adjuvants across human and veterinary applications. Alum, emulsion, CpG, and cytokine-based adjuvants, among others, are discussed from different perspectives and applications. The history and current knowledge of adjuvants were reviewed by (Xing et al.). The mechanisms and examples of commercial human vaccines were reviewed in their article. Yan et al. focused on cytokine-based adjuvants for animals, including interleukins, interferons, chemokines, and colony-stimulating factors. The authors describe delivery methods and carriers, along with a comprehensive synthesis of the different mechanisms of veterinary vaccine adjuvants. In a similar vein, human-derived adjuvants were discussed by Alrasheed et al. with a special focus on MERS-CoV vaccines. Molecules involved in antigen-presenting cell recruitment, immune activation, T cell differentiation, and memory cell development are potential adjuvants, and supporting evidence is provided. The authors propose testing several potential adjuvants in clinical trials to combat MERS-CoV infection, because some of them have already been evaluated for other infections.

Finally, Megdiche and Salerno-Gonçalves addressed a fundamental and emerging question: how do adjuvants shape the epigenetic landscape of immune responses? Epigenetics involves heritable modifications in gene expression. In this review, the authors provided insight into this topic and expanded on the potential role of adjuvants in cancer treatment. DNA methylation, histone modification, and non-coding RNAs modulate the potency of adjuvants on immune outcomes. Although several knowledge gaps remain, scientific evidence demonstrates that these outcomes can promote or inhibit responses. Remarkably, both responses can be desirable depending on the immune- preventive or therapeutic efficacy effects.

**Figure 1:**
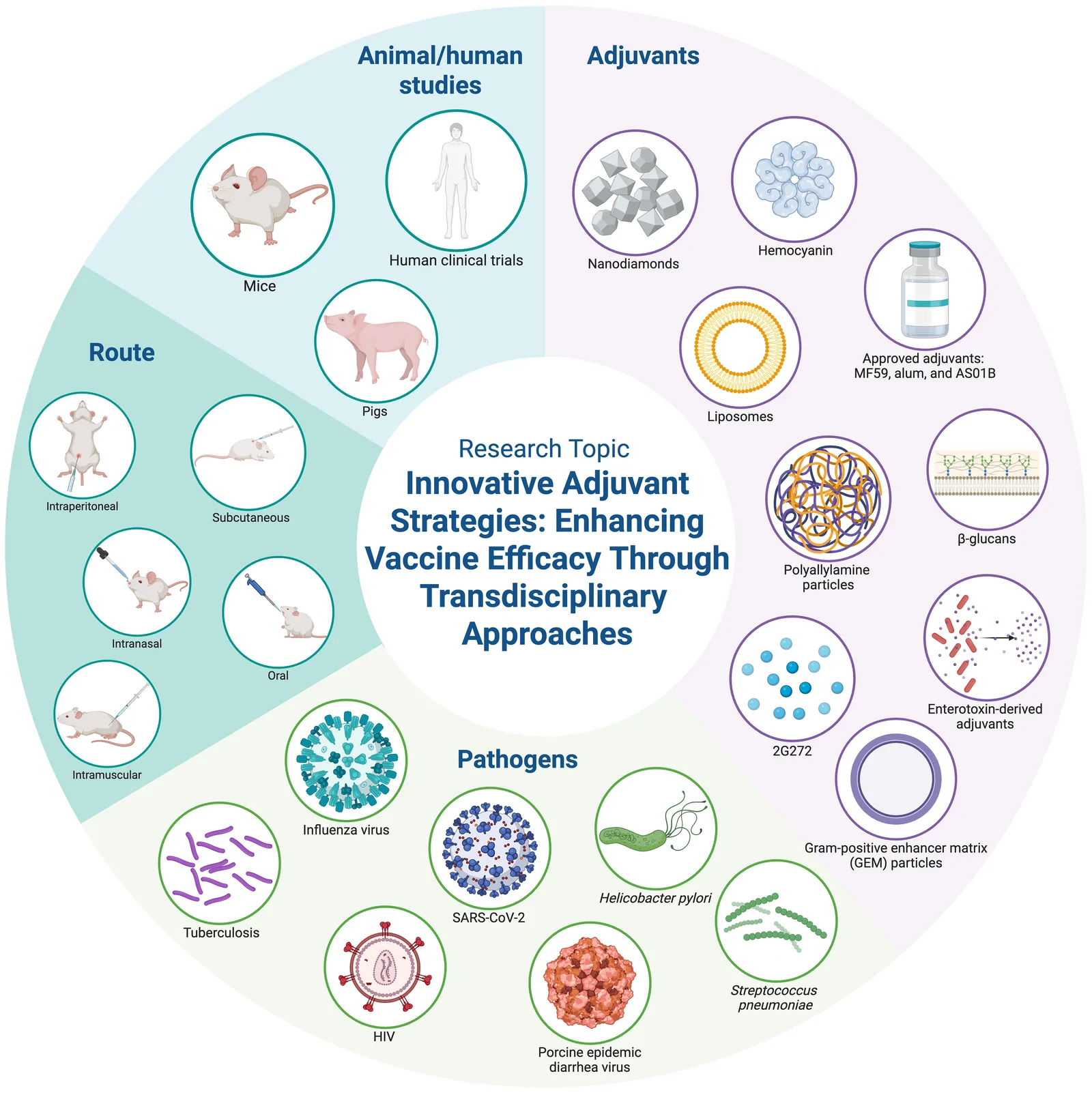
Illustrative synthesis of components for innovative adjuvant strategies that underscores this Research Topic.

Taken together, the studies featured in this Research Topic underscore the growing relevance and ongoing paradigm shift in adjuvant research (**Figure 1**), which has transitioned from the largely empirical approaches developed over the past century to a rational, transdisciplinary framework supported by data integration and artificial intelligence. In this context, the development of next-generation adjuvants will be pivotal to unlocking the full potential of innovative vaccine platforms.

